# Empowering heart failure patients: A mixed-method study on patient-centred nursing strategies to enhance self-care and daily functioning

**DOI:** 10.6026/973206300212716

**Published:** 2025-08-31

**Authors:** Pradeepa Govindaraj, Arul Subbaiah Arunachalam, Shankar Shanmugam Rajendran, Ananthi Duraikannu, Vasanth Pandian Thommai Antony Savari Muthu, Padmavathy Murugan, Athya Farheen Mohammed Sulthan

**Affiliations:** 1 Department of Medical Surgical Nursing, College of Nursing, Madras Medical College, Chennai, Tamil Nadu, India; 2 Department of Cardiology, Rajiv Gandhi Government General Hospital & Madras Medical College, Chennai, Tamil Nadu, India; 3 Department of Child Health Nursing, College of Nursing, Madras Medical College, Chennai, Tamil Nadu, India

**Keywords:** Patient-centred nursing strategies, heart failure, self-care ability, activities of daily living, mixed-method study, functional limitation

## Abstract

Heart failure patients face significant challenges in managing daily activities due to physical limitations, emotional stress, and inadequate post-discharge care. This study investigates the impact of patient-centred nursing strategies on self-care ability and activities of daily living (ADLs) in heart failure patients. A mixed-methods approach combining qualitative interviews and quantitative data from 60 patients showed significant improvements in self-care (58.13%) and ADL independence (66.67%) in the intervention group. The findings demonstrate that tailored nursing interventions effectively enhance patient outcomes, highlighting the importance of holistic, patient-centred care. This approach reduces dependency and promotes overall recovery.

## Background:

Heart failure is a life-altering condition that affects the heart's ability to pump blood efficiently, leading to symptoms like fatigue, breathlessness and reduced activity levels [1]. In India, the growing burden of heart failure is tied to rising rates of hypertension, diabetes and unhealthy lifestyle habits. Many patients, especially the elderly, experience severe physical limitations that impair their ability to perform daily tasks such as walking, bathing, or cooking [2]. Treatment alone is not enough, after hospital discharge, patients often return home without the necessary skills or confidence to manage their condition. Many rely on family members, increasing the caregiver burden and emotional stress [3]. Conventional care often focuses on medications and check-ups, but ignores the day-to-day challenges and emotional needs of both patients and families [4]. Patient centred nursing strategies help bridge this gap by offering tailored support, education, and simple interventions like breathing exercises, diet advice, and symptom monitoring [5]. These strategies aim not just to reduce physical symptoms, but also to restore confidence and independence in daily life. This study was born out of clinical experiences, where many heart failure patients were seen struggling with everyday activities despite regular treatment [6]. Therefore, it is of interest to examine whether structured, nurse-led care could reduce limitations, improve self-care ability, and enhance overall functioning among these patients.

## Methodology:

An exploratory sequential mixed-method design was adopted to assess the effectiveness of patient-centred nursing strategies on self-care ability and activities of daily living (ADL) among heart failure patients. The study was conducted at the Cardiology Department, Rajiv Gandhi Government General Hospital, Chennai, after ethical clearance and institutional approval. In the qualitative phase, five patients were selected via purposive sampling and interviewed using a semi-structured guide to explore physical limitations. Data were transcribed and analysed thematically using NVivo, which informed the intervention design. In the quantitative phase, 60 heart failure patients (EF < 40%, aged 40-70) were chosen by convenience sampling and divided into experimental and control groups (30 each). The experimental group received a 21-day structured nursing intervention covering medication, diet, fluid restriction, supervised exercise, and symptom monitoring. The control group received standard care. Data were collected using the Self-Care of Heart Failure Index (Riegel, 2009) and the Lawton-Brody ADL Scale (1969), and analysed using SPSS. A p-value ≤ 0.05 was considered statistically significant.

## Results:

Among the participants, 55% were male, and the largest age group was between 61-70 years (40%). Half of the sample had only primary education, and most were unemployed. Both experimental and control groups were statistically similar at baseline, with no significant demographic or clinical differences.

## Thematic content analysis yielded six main themes:

[1] Dependency in Daily Tasks

[2] Emotional Burden of Illness

[3] Challenges in Symptom Recognition

[4] Struggles with Medication Adherence

[5] Lack of Support Systems

[6] Role of Nurse in Empowerment

The comparison of self-care ability scores between the pretest and posttest, as shown in Table 1 (see PDF), reveals significant findings for the experimental group. The experimental group exhibited a considerable decrease in self-care ability, with a pretest mean score of 153.40 (SD = 26.24) and a posttest mean score of 93.80 (SD = 21.16), resulting in a mean difference of 59.60. The paired t-test result (t = 17.46, p = 0.001) indicates a statistically significant reduction in self-care ability, which is highly significant at the p ≤ 0.001 level. In contrast, the control group's self-care ability scores remained relatively stable, with a pretest mean of 155.10 (SD = 19.72) and a posttest mean of 151.93 (SD = 22.84), yielding a small mean difference of 3.17. The paired t-test result (t = 1.91, p = 0.06) suggests no significant difference in the self-care ability scores for the control group, indicating that the control group did not experience a statistically significant change. Similarly, Table 2 (see PDF) presents a comparison of the activity of daily living (ADL) scores between the pretest and posttest. The experimental group showed a notable improvement in ADL scores, with the pretest mean score of 1.57 (SD = 1.01) rising to a posttest mean of 3.93 (SD = 0.87), resulting in a mean difference of 2.36. The paired t-test result (t = 16.94, p = 0.001) indicates a highly significant improvement in ADL scores for the experimental group, with a very high level of statistical significance (p ≤ 0.001). In contrast, the control group exhibited only a minimal increase in ADL scores, from a pretest mean of 1.67 (SD = 0.99) to a posttest mean of 1.93 (SD = 0.87), producing a mean difference of 0.26. The paired t-test result (t = 1.77, p = 0.09) indicates no statistically significant change in ADL scores for the control group, demonstrating that the control group did not experience any meaningful improvement. In summary, the data from both tables highlight a significant improvement in self-care ability and ADL scores for the experimental group, while the control group showed no significant changes. These results underscore the positive impact of the intervention on the experimental group compared to the control group, with very high statistical significance observed in both self-care and ADL measures. A fair negative correlation (r = -0.38, P = 0.002) was found between self-care ability and ADL scores in the experimental group, suggesting that as self-care improved, dependency decreased. Better improvements were noted among participants aged 61-70, with normal BMI and non-smokers. These associations were statistically significant only in the experimental group.

## Discussion:

This study demonstrated that patient-centred nursing strategies significantly improved self-care ability and ADL among heart failure patients. These findings are consistent with Silva (2024) [6], who emphasised the role of structured self-care in enhancing patient outcomes. The observed improvements in functional status align with Kwame *et al.* (2024) [7] who highlighted the effectiveness of tailored rehabilitation in reducing ADL impairment. Additionally, Muthupillai *et al.* (2025) [8], who explored the lived psychological experiences of myocardial infarction patients, support the present study's emphasis on holistic, patient-centred care. Their phenomenological study revealed themes of fear, uncertainty, emotional vulnerability, and the need for individualized psychosocial support reinforcing the importance of empathetic nursing approaches in chronic cardiac conditions. The moderate negative correlation between self-care and ADL scores supports Wu *et al.* (2021) [9], indicating that better self-care reduces dependency. Associations with age, BMI and habits reflect patterns noted by Kontogianni (2010) *et al.* [10]. Overall, the study supports integrating nurse-led, patient-centred care into routine heart failure management for holistic recovery.

## Conclusion:

Individualized nursing interventions greatly enhanced self-care skills and daily functioning in heart failure patients. Incorporating these strategies into regular care can boost overall recovery and lessen dependence.
